# Retraction: Genetic Polymorphism of Interferon Regulatory Factor 5 (IRF5) Correlates with Allograft Acute Rejection of Liver Transplantation

**DOI:** 10.1371/journal.pone.0220815

**Published:** 2019-07-31

**Authors:** The PLOS ONE Editors

Concerns have been raised that the transplants performed in the local context at the time of procedures reported in this article [1] may have involved organs/tissues procured from prisoners [2].

Details as to the donor sources and methods of obtaining informed consent from donors were not reported in [1], and when following up on these concerns the authors did not clarify these issues or the cause(s) of donor death in response to journal inquiries. International ethics standards call for transparency in organ donor and transplantation programs and clear informed consent procedures including considerations to ensure that donors are not subject to coercion.

In addition, the authors did not provide documentation when requested by the journal to confirm that the study had institutional ethics approval, and the authors did not respond to inquiries about the availability of underlying data supporting this study.

Owing to the lack of documentation to demonstrate that this study had prospective ethical approval, insufficient reporting, unresolved concerns around the source of transplanted organs and whether they included organs from prisoners, and in compliance with international ethical standards for organ/tissue donation and transplantation, the *PLOS ONE* Editors retract this article.

The first author and corresponding author notified the journal that all authors disagree with the retraction. The other authors either could not be reached or did not respond directly.
